# Fabrication of Mesoporous Silica Shells on Solid Silica Spheres Using Anionic Surfactants and Their Potential Application in Controlling Drug Release

**DOI:** 10.3390/molecules171113199

**Published:** 2012-11-06

**Authors:** Ahmed Mohamed El-Toni, Aslam Khan, Mohamed Abbas Ibrahim, Mansour Al-Hoshan, Joselito Puzon Labis

**Affiliations:** 1 King Abdullah Institute for Nanotechnology, King Saud University, Riyadh 11451, Saudi Arabia; 2 Central Metallurgical Research and Development Institute, CMRDI, Helwan 11421, Cairo, Egypt; 3 Department of Pharmaceutics and Industrial Pharmacy, Faculty of Pharmacy, Al-Azhar University, Assiut 71111, Egypt

**Keywords:** core-shell, mesoporous, anionic surfactant, drug delivery

## Abstract

In this work, mesoporous shells were constructed on solid silica cores by employing anionic surfactante. A co-structure directing agent (CSDA) has assisted the electrostatic interaction between negatively charged silica particles and the negatively charged surfactant molecules. Synthetic parameters such as reaction time and temperature had a significant impact on the formation of mesoporous silica shelld and their textural properties such as surface area and pore volume. Core-mesoporous shell silica spheres were characterized by small angle X-ray scattering, transmission electron microscopy, and N_2_ adsorption–desorption analysis. The synthesized particles have a uniformly mesoporous shell of 34–65 nm and possess a surface area of ca. 7–324 m^2^/g, and pore volume of ca. 0.008–0.261 cc/g. The core-mesoporous shell silica spheres were loaded with ketoprofen drug molecules. The *in vitro* drug release study suggested that core-mesoporous shell silica spheres are a suitable nanocarrier for drug molecules offering the possibility of having control over their release rate.

## 1. Introduction

Nowadays, a lot of considerations have been paid to core/shell structures for their great versatility and the applications offered by the combined functionalities of cores and shells [1,2,3]. Amorphous mesoporous silica is an advantageous material in the field of materials science because it demonstrates excellent stability, tunable surface area and porosity and is non-cytotoxic [4]. Combining a mesoporous character with a core-shell structure is expected to provide a new generation of functional materials with improved and tailorable properties [5,6]. In that regards, mesoporous shell-based nano-structures have shown a wide variety of useful applications, including drug delivery [7], catalysis [8], water treatment [9], and protein separation [10].

Mesoporous silica shell formation has been extensively done either by using a cationic surfactant, cetyltrimethylammonium bromide (CTAB), as a template to create ordered mesopores [11] or by using *n*-octadecyltrimethoxysiliane (C18-TMS) for obtaining disordered ones [12]. Zhang *et al.* have also developed “surface-protected etching” for formation of non-ordered mesoporous silica shell by using poly(vinyl pyrrolidone) (PVP) as the outer surface protector, together with NaOH [13] as etching agent.

Recently, Che and Tatsumi have reported a novel approach for the fabrication of highly ordered mesoporous silica materials with anionic surfactants and a co-structure directing agent (CSDA) [14]. In their approach, aminosilane (CSDA), having a positively charged amine, interacts electrostatically with the anionic surfactant micelles. Simultaneously, the alkoxysilane sites of CSDA co-condense with the inorganic precursors. This approach has provided a series of novel mesostructured phases, such as lamellar and hexagonal mesostructures [15,16], as well as well-defined morphologies [17]. In addition, mesoporous silica synthesized using anionic surfactant and CSDA would be beneficial to the preparation of surface amino-functionalized mesoporous silica after simple removal of the anionic surfactant by an acid-extraction step [18]. An advantage of constructing a mesoporous silica shell using an anionic surfactant around silica cores, is that it will be a platform for synthesizing other core-mesoporous shell structures including metal, magnetic and quantum dot-mesopororus silica shell nanoparticles. To achieve targeted drug delivery, we could have magnetic core-mesoporous shell structures that cannot be effectively assembled by conventional mesoporous nanoparticles. Other advantages for core-mesoporous shell structures over conventional mesoporous silica, is the possibility of having multi-functionality integrated into single nanoparticles together with monodispersity, since conventional mesoporous silica nanoparticles cannot provide multi-functionality as a core-mesoporous shell structure does, besides the difficulty to have monodispersity. In addition, an amino functionalized (due to the usage of CSDA) radially oriented mesoporous silica shell can be considered as ideal cavity for versatile applications like drug delivery and catalysis.

Herein, we report the fabrication of a mesoporous silica shell on solid silica cores by using an anionic surfactant and CSDA. The synthetic parameters were optimized to provide uniform mesoporous silica shells with monomodal pore size distributions. The variation of textural properties with changing the synthesis conditions was observed. Finally, the drug control release behavior for the core-mesoporous shell silica spheres is presented.

## 2. Results and Discussion

### 2.1. Effect of Synthesis Route

**Figure 1:**
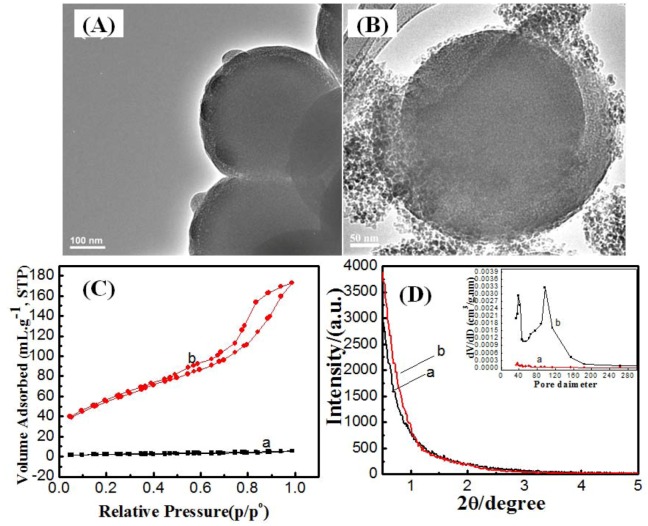
TEM images for mesoporous silica shell constructed on silica cores by (**A**) one-pot synthesis and (**B**) two-pot synthesis; (**C**) N_2_ adsorption/desorption isotherms and (**D**) low X-ray diffraction patterns for mesoporous silica shell constructed on silica cores by (**a**) one-pot synthesis and (**b**) two-pot synthesis and pore-size distribution from the adsorption branch (inset).

The construction of mesoporous silica shells on solid silica cores can be performed by either a one or two-pot synthesis route. In the one-pot route, after the solid silica cores were formed, the co-structure directing agent, surfactant and silica source were added to the formed cores in the same pot. In the two-pot synthesis, first, silica cores are prepared and centrifuged to remove ethanol. In the second step, the co-structure directing agent, surfactant and silica source were added to a silica cores/ water dispersion. The one-pot synthesis route results in the formation of non-porous shells (Figure 1A). Random pores can be seen in silica shell which was covered by another dense silica layer. On the other hand, the two-pot synthesis route allows the formation of mesoporous silica shells on solid silica cores (Figure 1B). In addition, small silica nuclei [19] were formed as by-product. However, the main difference between one and two-pot synthesis route is the presence of ethanol in the one-pot synthesis. At the presence of ethanol, *N*-lauroylsarcosine sodium molecules seem to interact more strongly with ethanol rather than with water which raises the critical micelle concentration value and thus suppresses micellization. These results are in an agreement with the findings of Wang *et al.* [20] who have reported mesophase regression when the ethanol to surfactant ratio exceeded 73.8.

The nitrogen adsorption/desorption isotherms measured at 77 K for the calcined samples resulting from the one and two-pot syntheses are shown in Figure 1C. The isotherm for the one-pot synthesis product is characteristic of a non-porous material with a total pore volume of 0.008 cc·g^−1^, as shown in Table 1. The low pore volume could be attributed to the random pore coverage (Figure 1A) in the silica shell with a further dense silica layer. On the other hand, the isotherms for the two-pot synthesis product exhibited the type IV isotherm characteristic of mesoporous materials with a total pore volume of 0.267 cc·g^−1^. The isotherms show two major capillary condensation steps in the relative pressure ranges 0.1–0.7 and 0.80–0.95, implying two sets of mesopores, respectively, of ~3.6 nm and ~10 nm in diameter [21]. The small pores can be attributed to anionic surfactant micelles. The large pores could be due to the voids between the agglomerated silica nuclei. The BET surface area for core- mesoporous shell samples prepared by one and two-pot synthesis was 7 and 199.6 m^2^/g, respectively as shown in Table 1. The powder X-ray diffraction (XRD) patterns for the calcined samples of one and two-pot syntheses product are shown in Figure 1D. Both samples do not show any peaks at low angle due to the loss of long-range order of the mesopores. However, despite the fact that the mesoporous shell was formed in a two-pot synthesis route, nevertheless these pores did not pack in ordered arrangements as reflected from small-angle X-ray diffraction.

**Table 1:** Synthesis conditions and textural parameters for core-mesoporous shell silica spheres prepared by anionic surfactant.

Sample code	Synthesis route	Synthesis temperature/°C	Synthesis time	Surface area/cm^2^·g^−1^	Pore volume/cc·g^−1^	Shell thickness/nm		Pore size/nm
						T_TEM_	T_DLS_	
SCMS-1	One pot	25	20 h	7	0.008	34	38	3.6, 4, 6
SCMS-2	Two pot	25	20 h	199	0.207	41	43	3.6, 10
SCMS-3	Two pot	50	20 h	191	0.234	58	57	3.6, 10
SCMS-4	Two pot	80	20 h	286	0.243	65	71	3.6
SCMS-5	Two pot	80	6 h	324	0.261	50	53	3.6
SCMS-6	Two pot	80	2 h	226	0.184	42	46	3.6
Hexa-an	-	25	20 h	332	0.354	-	-	3.6

### 2.2. Effect of Reaction Temperature

**Figure 2:**
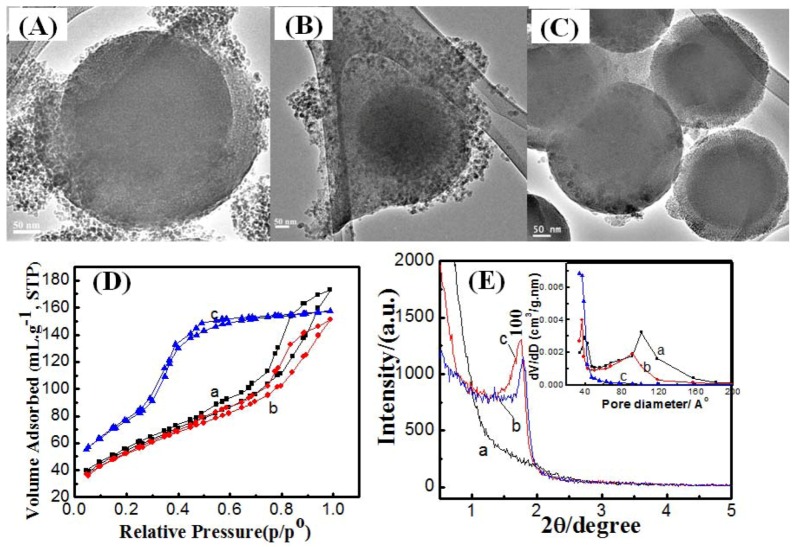
TEM images for mesoporous silica shell constructed on silica cores by two-pot route at synthesis temperature (**A**) 25; (**B**) 50 and (**C**) 80 °C at 20h reaction time; (**D**) N^2^ adsorption/desorption isotherms and (**E**) low X-ray diffraction patterns for mesoporous silica shell constructed on silica cores by two-pot route at synthesis temperature (**a**) 25, (**b**) 50 and (**c**) 80 °C at 20h reaction time. Inset is the pore size distribution calculated from adsorption branch.

Variation of coating temperature was quite effective in reducing the formation of secondary silica nuclei and building more homogenous mesoporous silica shells. The TEM images in Figure 2A show that at 25 °C secondary silica nuclei were also formed as a by-product around the core-mesoporous shell silica spheres. Further elevation of reaction temperate slightly reduced the formation of secondary silica particles, as seen from Figure 2B, but the mesoporous shell was not homogenously surrounding the silica cores. However, performing synthesis at 80 °C effectively suppressed secondary silica nuclei and increased the silica shell thickness.

The adsorption-desorption isotherms show two major capillary condensation steps, for samples prepared at 25 and 50 °C, corresponding to 3.6 nm and 10 nm mesopores, respectively. The bi-model pore size distribution can be also confirmed from the inset of Figure 2D. Increasing the synthesis temperature caused the suppression of larger size pores (10 nm) by suppressing the formation of secondary silica nuclei. The specific surface area of the solid core-mesoporous shells was found to be 199, 191, and 286 m^2^/g at reaction temperatures of 25, 50, 80 °C, respectively. On the other hand, the total pore volume ranged from 0.207, 0.234 to 0.243 cc/g at temperatures of 25, 50, 80 °C, respectively. It is worth indicating that the textural properties, surface area and pore volume, were enhanced by increasing the reaction temperature. The low-angle X-ray diffraction shows that at 25 °C, no diffraction peaks were noticed which indicates the absence of long range ordering of mesopores. However, increasing the temperature resulted in the appearance of the (100) peak which is characteristic of hexagonal mesophases. The weakening of other hexagonal reflections, (110) or (200), can be attributed to the distortion, to a certain extent, from a perfect 2D hexagonal mesostructure, due to the packing of the radially oriented mesopores in the spherical shell [22]. These results suggested that increasing the reaction temperature helps in the formation of mesoporous shells with 2D hexagonal ordering. The reaction temperature has a significant impact on the formation of mesoporous silica, because temperature affects the thermodynamics of the liquid–crystal phases of the surfactant and the kinetics of the hydrolysis and condensation of TEOS [23]. It is expected that increasing synthesis temperature would allow the dissolution of secondary silica nuclei and their re-precipitation into the mesoporous shell. The disappearance of secondary silica nuclei from TEM images with increasing synthesis temperature suggested this explanation. 

### 2.3. Effect of Reaction Time

**Figure 3:**
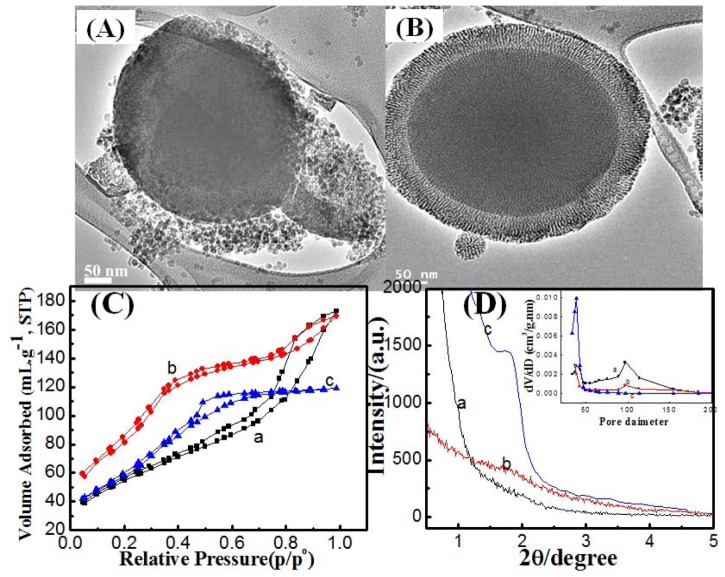
TEM images for mesoporous silica shell constructed on silica cores by two-pot route at synthesis time (**A**) 6h and (**B**) 2h at 50 °C; (**C**) N_2_ adsorption/desorption isotherms and (**D**) low X-ray diffraction patterns for mesoporous silica shell constructed on silica cores by two-pot route at synthesis time (**a**) 20, (**b**) 6 and (**c**) 2h at 50 °C and pore-size distribution from the adsorption branch (inset).

Another tool for suppression of secondary silica nuclei formation and improvement of mesoporous shell homogeneity is the optimization of reaction time of the coating step in the two-pot synthesis route. From the TEM images (Figure 3), decreasing reaction times helped in suppression of silica nuclei. At 2 h reaction time, a complete and homogenous mesoporous silica shell can be seen formed around solid silica cores. However, shortening of the reaction time from 20, 6 to 2 h resulted in slight decreases of silica shell thickness from 65, 50 to 42 nm (Table 1), respectively. 

The adsorption-desorption isotherms showed the presence of two condensation stages, at 0.1–0.7 and 0.80–0.95, at 20 and 6 h reaction times, which suggest the formation of bi-model mesopores. However, shortening the reaction time to 2 h causes the formation of a hysteresis loop with only one stage condensation which is characteristic for mono-model mesoporous silica. In other words, 2 h reaction time leads to suppression of large size void pores by preventing secondary silica nuclei formation. It can be seen from Table 1 that the textural properties tended to decrease with shortening reaction time, which can be attributed to a decline of silica shell thickness. The observation of a triangular hysteresis loop (type IV hysteresis) in the 2 h sample can be attributed to some defects and non-uniformity within the mesochannels [24]. Low-angle X-ray diffraction shows that at 2 h reaction time, the (100) peak, which is characteristic of hexagonal mesopores, was observed. This peak begins to decay with increasing reaction time. Elongation of reaction time causes the loss of ordering due to dissolution-precipitation equilibriums that lead to the dissolution of part of mesoporous silica shell to form secondary silica nuclei. This dissolution and precipitation are usually caused by the presence of hydroxyl ions that were adsorbed on silica spheres during the silica core synthesis step [25]. Therefore, 2 h is quite enough to provide an ordered mesoporous silica shell.

**Figure 4:**
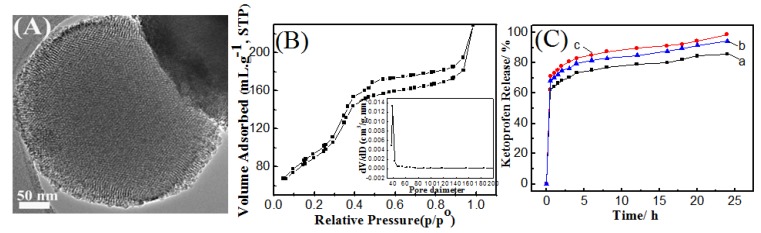
(**A**) TEM image; (**B**) N_2_ adsorption/desorption isotherm for hexagonal mesoporous silica nanoparticles synthesized by anionic surfactant and (**C**) Cumulative drug release from (**a**) hexagonal mesoporous silica nanoparticles synthesized by anionic surfactant and the solid core- mesoporous shell silica samples prepared by two-pot synthesis at (**b**) 80 and (**c**) 25 °C in simulated body fluid. Pore-size distribution for hexagonal mesoporous silica nanoparticles (inset).

For comparison purposes, hexagonal mesoporous silica nanoparticles were prepared as shown in Figure 4A. It is clear that round silica spheres with hexagonal pore ordering can be seen. Hexagonal mesoporous nanoparticles showed superior total pore volume as compared with SCMS samples (Figure 4B and Table 1). On the other hand, its surface area was closer to that of the SCMS-5 sample. However, the hexa-an sample possessed a monomodel pore size distribution with diameter of 3.6 nm. To explore the capability of solid core-mesoporous shell silica nanospheres as drug carriers, ketoprofen, a typical anti-inflammatory drug, was introduced into the pores of core-mesoporous shell silica spheres samples prepared at 25 and 80 °C. The uptake amount of ketoprofen is ca. 21.33, 29.68 wt%, respectively. On the hand, ketoprofen uptake amount in hexagonal mesoporous nanoparticles was 33.95 wt%. The high drug loading capacity of SCMS-4 (80 °C) and hexa-an samples can be attributed to the large pore volume beside shell thickness (only in case of the SCMS sample). Shell thickness represents the length of mesopore in which ketoprofen molecules can be stored. However, in hexagonal mesoporous nanoparticles, drug molecules will diffuse out from hexagonal mesopores much slower due to its large pore volume. The release behavior of ketoprofen in a simulated body fluid (SBF) was relatively fast during the first 2 h, and then more controlled release took place to reach the value of 86.07, 98.96 and 94.31% after 24 h for hexa-an, SCMS-4 and SCMS-2 samples, respectively. The fast initial release could be due to the rapid leaching of free ketoprofen molecules from the pore entrances. Thereafter, ketoprofen continues to dissolve slowly into the liquid phase as the solvent fills the capillary and diffuses from the system out of mesochannels and hexagonal mesopores. Thus, the mesochannel length must be considered as crucial factor that affects the drug-release rate [26,27]. Compared to hexa-an with superior large pore volume, core-mesoporous shell silica spheres showed promising drug release behavior.

## 3. Experimental

### 3.1. Chemicals

*N*-lauroylsarcosine sodium (Sar-Na), 3-aminopropyltrimethoxysilane (APMS), polyvinylpyrrolidone (PVP) average Mw ~29,000 and tetraethoxysilane (TEOS) were purchased from Sigma-Aldrich (Seelze, Germany). All the chemical reagents were used without further purification.

### 3.2. Synthesis

#### 3.2.1. Synthesis of Solid Silica Cores

Aqueous ammonia (0.875 mL) was added to a solution containing ethanol (18 mL) and deionized water (2.6 mL), followed by the addition of TEOS (1.5 mL) to the solution with vigorous stirring. The resulting mixture was then heated at 30 °C for 1 h and the silica precipitate was collected by centrifugation and washed three times with water. The molar composition of the suspension was as follows: TEOS:EtOH:NH_3_:H_2_O = 1:45.8:3.3:21.5. In order to form the mesoporous silica shell around the above prepared silica particles, we adopted two different strategies consisting of either one or two-pot synthesis routes.

#### 3.2.2. One-Pot Synthesis Route

In the one-pot synthesis route, water (10 mL) and 3-aminopropyltrimethoxysilane (APMS, 0.10 mL) were added respectively to the above solution where the mixture was stirred for 30 min. Then a solution of *N*-Lauroylsarcosine sodium (0.2933 g, 1 mmol) in H_2_O (25 mL), which already has been acidified with 0.1 M HCl (4 mL) was added to the above solution and stirred for further 1 h. Finally TEOS (1.5 mL) was added and the mixture kept under stirring at 50 °C for 2 h.

#### 3.2.3. Two-Pot Synthesis Route

In the two-pot synthesis route, solid silica cores were prepared as mentioned above. The silica precipitate was collected by centrifugation and washed three times with water to remove ethanol and reaction by-products. To construct the mesoporous silica shells, SiO_2_ particles (0.3 g) were dispersed in H_2_O (15 mL) by ultrasonication for 10 min. Thereafter, APMS, and *N*-lauroylsarcosine sodium acidified solution and TEOS were added, respectively, to the reaction mixture identical to the one-pot synthesis route with subsequent stirring at 50 °C for 2 h. The final solid was recovered by centrifugation (10,000 rpm, 10 min), washed with deionized water for three times and dried in an oven at 60 °C for 12 h. Template removal was done by heat-treatment in an air stream at 550 °C for 6 h.

For comparison, mesoporous silica nanoparticles were prepared using anionic surfactant by dissolving *N*-Lauroylsarcosine sodium (0.2933 g, 1 mmol) in H_2_O (25 mL), then acidifying with 0.1 M HCl (4 mL), with stirring for further 1 h. Thereafter, 3-aminopropyltrimethoxysilane (APMS, 0.1 mL) and TEOS (1.5 mL) were added, respectively with continuous stirring for 20 h at 25 °C. This hexagonal mesoporous silica sample was denoted as hexa-an as shown in Table 1.

#### 3.2.4. *In-Vitro* Drug Storage Study

Core-mesoporous shell silica samples prepared at 25 and 80 °C (50 mg) were added into 50 mg/mL ketoprofen-ethanol solution. The suspension was stirred for 2 h while the evaporation of ethanol was prevented. Then the sample-drug suspension was separated by high-speed centrifugation and dried in a vacuum oven at 60 °C. Filtrate (1.0 mL) was extracted with a vial and diluted to 100 mL, and then was analyzed by UV/Vis spectroscopy (Shimadzu UV-2550, Tokyo, Japan) at a wavelength of 265 nm. The calibration curve of ketoprofen was determined by taking absorbance *versus* ketoprofen concentration between 0 and 200 mg/mL as parameters.

#### 3.2.5. *In Vitro* Drug Release Study

Core-mesoporous shell silica samples were separately immersed in simulated body fluid (SBF) at 37 °C, with stirring at a rate of 100 rpm. Then, release medium (2.0 mL) was removed for analysis at given intervals with a syringe, and the same volume of fresh release medium was injected. The extracted medium was diluted to a desired concentration with simulated body fluid, and analyzed by UV/Vis spectroscopy at a wavelength of 265 nm.

### 3.3. Characterization

TEM analysis was performed using a JEOL JSM-2100F Electron microscope (Tokyo, Japan) operated at 200 kV. The average silica shell thickness was roughly estimated by measuring the thickness of the silica layer formed on 20 core-mesoporous shell spheres using a transmission electron microscope and denoted as T_TEM_. Particle size distributions of the solid silica cores (Tcore) and solid core-mesoporous shell (T_core-shell_) spheres were measured by dynamic light scattering (DLS) on a Malvern Nanosizer ZS instrument (Malvern, Worcestershire, UK). The shell thickness was also obtained by subtracting T_core-shell_ − T_core_ as T_DLS_. Shell thickness obtained by TEM observation gives more precise results than DLS one. However, both values T_TEM_ and T_DLS_ were added to Table 1. Powder X-ray diffraction (XRD) patterns were recorded on a PANalytical X'Pert PRO MPD (Lelyweg, The Netherlands) with Ni-filtered Cu KR radiation (45 kV, 40 mA). Nitrogen sorption isotherms were measured at 77 K with a Quantachrome NOVA 4200 analyzer (Boynton Beach, Fla USA). Before measurements, the samples were degassed in a vacuum at 200 °C for at least 18 h. The Brunauer-Emmett-Teller (BET) method was utilized to calculate the specific surface areas (SBET) using adsorption data in a relative pressure range from 0.05 to 0.35. By using the Barrett-Joyner-Halenda (BJH) model, the pore volumes and pore size distributions were derived from the adsorption branches of isotherms and the total pore volumes (Vt) were estimated from the adsorbed amount at a relative pressure P/P0 of 0.992. The UV/Vis absorbance spectra were measured with a Shimadzu (Tokyo, Japan) UV-2550 UV-Vis Spectrophotometer.

## 4. Conclusions

Mesoporous silica shells were constructed on solid silica cores using an anionic surfactant and CSDA. A two-pot synthesis route allowed the formation of mesoporous silica shells around solid silica cores due to the absence of ethanol which elevates the critical micelles concentration of the anionic surfactant. Optimization of synthesis parameters such as synthesis temperature and time suppresses the formation of secondary silica nuclei that is responsible for formation of 10 nm mesopores and allows formation of homogenous silica shells. An *in vitro* ketoprofen release study suggested that core-mesoporous shell silica spheres had reasonable drug storage capacity with slow release behavior.
